# Virological treatment failure and associated factors among adults on first-line antiretroviral therapy in West Hararghe, Ethiopia

**DOI:** 10.3389/fpubh.2025.1440504

**Published:** 2025-06-02

**Authors:** Ebisa Zerihun, Kenesa Tesema, Fekadu Abera

**Affiliations:** ^1^Department of Nursing, College of Health Science, Oda Bultum University, Chiro, Ethiopia; ^2^Faculty of Health Science, Rift Valley University, Adama, Ethiopia

**Keywords:** HIV/AIDS, virological failure, first-line ART, early ART initiation, West Hararghe

## Abstract

**Background:**

Virological failure on first-line antiretroviral therapy (ART) remains a major challenge in the management of HIV/AIDS in resource-limited settings, including Ethiopia. However, the prevalence of virological failure and its associated factors among adult patients on first-line ART in West Hararghe, Ethiopia, are not well documented. Therefore, this study aimed to assess virological treatment failure and its determinants among people living with HIV (PWH) in West Hararghe, Eastern Ethiopia.

**Methods:**

A retrospective cohort study was conducted using routine HIV-related data from a health facility providing services in West Hararghe between 01 January 2017 and 31 December 2020. Sociodemographic, behavioral, clinical, and HIV-related data were collected through medical chart reviews. Virological treatment failure was defined as a plasma viral load above 1,000 copies/mL based on two consecutive viral load measurements. A logistic regression model was used to identify factors associated with virological treatment failure.

**Results:**

A total of 257 records of PWH were reviewed and included in this analysis. Of these, 11.67% experienced virological failure while on first-line ART. Baseline undernutrition (AOR = 3.717: 1.051, 13.139), non-disclosure of serostatus (AOR = 4.453: 1.340, 14.793), early (≤ 30 days) ART initiation (AOR = 0.235: 0.064, 0.859), a history of missed daily ART doses (AOR = 3.156: 1.007, 9.891), and the use of a dolutegravir (DTG)-based regimen (AOR = 0.275: 0. 085, 0.895) were statistically associated with virological failure on first-line ART.

**Conclusion:**

Virological failure on first-line ART was found to be significantly high in West Hararghe. Factors such as undernutrition, non-disclosure of serostatus, interruption of ART doses, and the use of DTG-based regimens were identified as significant predictors of virological treatment failure. Healthcare providers should focus on the accelerated initiation of ART (preferably with a DTG-based regimen) and supplemental nutritional therapy for patients with undernutrition.

## 1 Introduction

The primary goal of antiretroviral therapy (ART) is to suppress viral replication, increase patients’ survival rate through the reduction of HIV transmission, prevent HIV-related illness, avert AIDS-related deaths, help patients live a normal lifespan, and enhance both health and economic outcomes (1–3). Patients on ART are recommended to undergo periodic viral load monitoring to ensure effective and durable treatment outcomes (4, 5). Viral load monitoring has become the standard of care for detecting ART failure and has been explicitly recommended in international guidelines (2, 6).

The World Health Organization (WHO) recommends isoniazid (INH) and cotrimoxazole preventive therapy (CPT) to reduce morbidity and mortality associated with opportunistic infections in individuals living with HIV. INH is recommended for individuals with a positive tuberculin skin Test (TST) and a negative chest X-ray to prevent the risk of developing active TB. CPT is recommended for individuals with a CD4 count below 200 cells/mm^3^ to protect against opportunistic infections such as Pneumocystis pneumonia and bacterial infections (7). Research indicates that CPT can reduce mortality by up to 60% when initiated alongside antiretroviral therapy (ART), particularly in patients with a low CD4 count (8).

According to Ethiopia’s national guidelines, a viral load of more than 1,000 copies/mL in a patient who has been on ART for at least 6 months indicates either therapeutic failure due to antiretroviral resistance or poor adherence to treatment (9). Patients whose viral loads are not suppressed at retesting after adherence support for 3–6 months are classified as having virological failure due to probable drug resistance and are switched to second-line therapy (3, 9).

Before 2016, virological monitoring was rarely conducted in Ethiopia because of limited access to viral load testing facilities. As a result, a great deal of research conducted in Ethiopia focused on the survival outcome before the scale-up of regular viral load services, when clinical and immunological assessments were the primary methods for diagnosing treatment failure (10–13).

Studies conducted in Ethiopia have reported varying rates of virological failure, ranging from 1 to 9 per 100 person-years, with proportions ranging from 11 to 28% of patients failing treatment (14–18). However, the prevalence of first-line ART failure and its associated factors among people living with HIV (PWH) in West Hararghe, Ethiopia, are not well understood. This region experiences distinct socioeconomic practices and a relatively high HIV prevalence following the implementation of routine viral load monitoring.

Previous Ethiopian studies used clinical and immunologic criteria to assess factors influencing treatment failure, whereas we propose the identification of patient characteristics to determine risk factors associated with treatment failure (19, 20).

**Figure 1:**
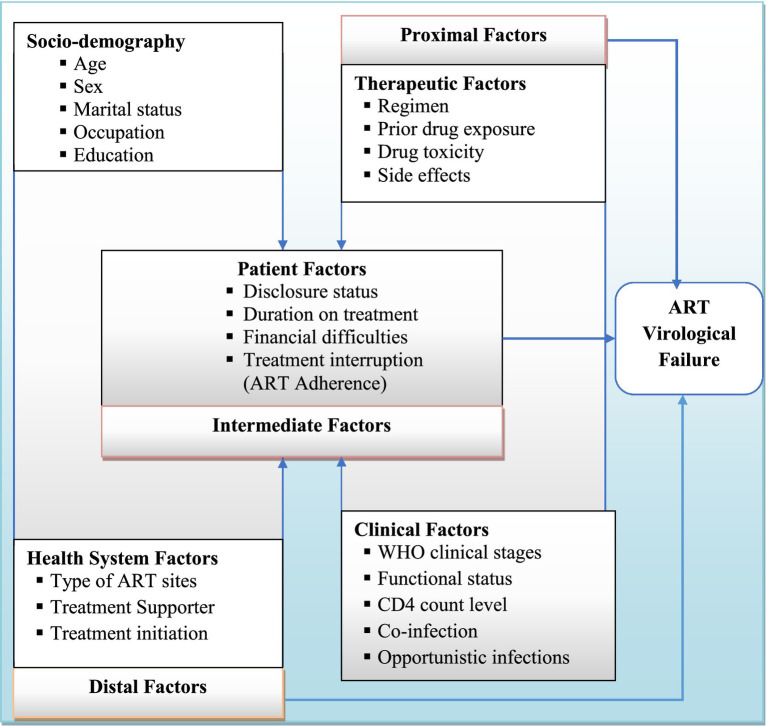
Conceptual framework describing predictors of ART virological treatment failure [Source: developed by the investigators, combining variables from the literature review (10–13, 19)].

A clear distinction between participants with and without ART failure is important for identifying predictors of treatment failure using virological (plasma viral load) criteria (6). Understanding the prevalence of virological failure and its determinants is crucial for early prevention and improving the quality of life for HIV patients on first-line ART. However, virological failure and its associated factors among adult patients on first-line ART in West Hararghe are not well documented. Therefore, this study aimed to determine the prevalence of virological failure and its determinants among PWH in West Hararghe, Eastern Ethiopia. Identifying these determinants will allow us to develop or improve interventions that will ultimately improve HIV treatment outcomes among adults in this region (Figure 1).

## 2 Materials and methods

### 2.1 Study area and period

The study was conducted at an ART site in the West Hararghe zone from January 2017 to December 2020. The West Hararghe zone is located in the eastern part of Ethiopia, 316 km from Addis Ababa, the capital city of Ethiopia. Based on the 2007 Census conducted by the Central Statistical Agency (CSA), this zone had a total population of 1,871,706, an increase of 47.16% since 1994. According to the West Hararghe Zone Administration Health Information System 2018 annual report, approximately 1,605 adults were newly started on ART during the 4-year data retrieval period (West Hararghe Zone Health Bureau Office, HIV Prevention and Control Unit, 2020). Free access to ART began in 2005 in West Hararghe, where there are currently 14 primary care health facilities and two general hospitals public hospitals providing this service.

According to the 2018 Ethiopian ART guidelines, the preferred first-line regimen for adults is a once-daily dose of tenofovir (TDF) and lamivudine (3TC) combined with dolutegravir (DTG) or efavirenz (EFV) (TDF + 3TC + DTG or TDF + 3TC + EFV,) depending on the patient’s clinical conditions (21). Since Aug 2016, West Hararghe has implemented routine viral load monitoring with enhanced adherence counseling (EAC) for individuals with a detectable viral load, using referral testing services. The study was conducted among adults who initiated first-line ART between 01 January 2017 and 31 December 2020 at Chiro General Hospital, West Hararghe. The data for this study were collected between 15 December 2021 and 31 December 2021.

### 2.2 Study design

A retrospective cohort study design was used.

### 2.3 Population

Adults aged 15 years and above who initiated first-line ART in the West Hararghe zone were potential study participants. The actual study participants were randomly selected adults aged 15 years and above who initiated first-line ART during the data collection period and met the inclusion criteria.

### 2.4 Inclusion and exclusion criteria

The study included all individuals who had completed at least 6–12 months of follow-up after starting first-line ART and had available viral load evaluation results. Since virological failure is defined based on viral load measurements taken at least 6–12 months after ART initiation, the observational window was extended to capture the second viral load result for the participants who underwent extended EAC (21). To meet this definition, the participants were required to have undergone at least two viral load evaluations following 6 months on ART. The eligibility criteria for ART initiation were based on the Ethiopian national guidelines. These guidelines recommend initiating ART for all HIV-infected individuals regardless of the CD4 cell count. Only individuals with complete viral load data who met the inclusion criteria were included in the outcome analysis. Patients with incomplete viral load data and those who started ART at another site (with incomplete baseline data recording) were excluded. Incomplete viral load data were defined as having fewer than two viral load measurements during the follow-up period.

### 2.5 Sample size determination and sampling technique

#### 2.5.1 Sample size determination

The sample size was determined using EpiInfo 7 version 3.5.3, based on factors associated with virological failure from a previous Ethiopian study. A double population proportion assumption was applied for a two-sided hypothesis test with a 95% significance level, 80% power, and an equal number of exposed and unexposed groups. Among the risk factors reported in a study conducted in Northeast Ethiopia, CD4 status (CD4 level < 500 cells/mL compared to > 500 cells/mL) of patients (AOR = 4.78) (22) yielded the maximum sample size. A detailed summary of the sample size calculation for the risk factors is provided in Supplementary Table 1. The final intended maximum sample size for this study was 272, with an additional 20% contingency to account for potential participant dropout or data loss.

#### 2.5.2 Sampling procedure and sampling technique

Among the 876 PWH receiving care at Chiro General Hospital, 272 patient records were randomly selected based on the year of ART initiation.

The sample size was proportionally allocated to each year, depending on the actual number of new adults initiated on ART during that specific year. The sampling frame was created using patient registers and electronic databases, based on the years of ART initiation. Finally, a simple random sampling technique was used to select a proportionate number of patient records.

### 2.6 Data collection tool and procedure

Data were collected by reviewing the patients’ medical records using an extraction tool prepared in English. A structured record review checklist was developed based on the data elements from the nationally standardized HIV patient intake and follow-up formats, which were created according to the WHO patient monitoring guidelines. Peer-reviewed published literature (10, 13, 23) was used to collect data from the ART register, patient’s cards, and ART intake forms.

Health professionals who were trained in comprehensive HIV care were selected as supervisors and data collectors to ensure the quality of the data. Health Information and Communication Technology (HICT) staff, assigned to manage the data for this study, worked at different sites. The supervisors collected the checklists after ensuring their completeness and consistency; if they found any problems, they cross-checked the data against the source. All missing data from the patients’ medical records were cross-checked with the ART electronic database and laboratory registers.

### 2.7 Study variables

#### 2.7.1 Dependent variables

The primary outcome of interest was virological failure, which was defined as a persistently detectable viral load of greater than or equal to 1,000 copies/mL, based on two consecutive measurements within a three-month interval, after at least 6 months of ART.

#### 2.7.2 Independent variables

The main exposure was ART adherence, which the WHO defines as the extent to which a person’s behavior corresponds with the agreed-upon recommendations from a healthcare provider (3). Other exposure variables extracted from the patients’ baseline and follow-up records included exposure characteristics measured at ART initiation—such as age, sex, marital status, education, occupation, disclosure, availability of a caretaker, type of facility, and the duration of ART since confirmation of HIV positive. Baseline and follow-up characteristics included body mass index (BMI), functional status, WHO clinical stage, CD4 level, opportunistic infections, comorbidities, duration on ART, reported side effects, drug toxicity, first-line ART substitutions, missed ART doses, and treatment adherence.

### 2.8 Operational definitions

#### 2.8.1 Virological ART treatment failure

According to the WHO (6, 9), virological ART treatment failure is defined as a second viral load measurement greater than 1,000 copies/mL, taken 3 months after an initial detectable viral load in a patient who has been on ART for at least 6 months. Therefore, the patients with a second viral load result greater than 1,000 copies/mL were categorized as having virological treatment failure, whereas those with a second viral load result less than 1,000 copies/mL were categorized as having virological treatment suppression.

#### 2.8.2 Treatment adherence

This was measured as the percentage of missed doses within a month, assessed through the remaining pill count or patient self-report. Clinicians consider good, fair, and poor adherence if the percentage of missed dose is > 95%, 85–94, and <85%, respectively, (21).

#### 2.8.3 Functional status

It was defined as working, capable of going out of home and performing routine activities; ambulatory, capable of self-care and going to the toilet without assistance; and bedridden, unable to go to the toilet without assistance (9).

#### 2.8.4 Treatment supporters

This refers to a specific individual who provides emotional, social, or practical support related to HIV treatment.

### 2.9 Data quality management

To ensure the quality of the data, different mechanisms were used, including the careful design of the data extraction tool, pretesting the checklist, appropriate recruitment, orientation and assignment of data collectors, and follow-up with data collectors and supervisors. Data clerks and case managers assisted the data collectors by identifying patient records. The investigators and supervisors checked the collected data for completeness, and corrective measures were taken accordingly, including cross-checking with the data source. The collected data were entered, coded, cleaned, edited, and explored before analysis.

### 2.10 Data processing and analysis

A standard coding guide, data entry procedures, and detailed computer editing specifications were prepared to ensure consistency in the data management process. The data were entered into the computer using the EpiData version 3.02 software and then exported to STATA 15 for cleaning and analysis. Univariable analyses such as frequencies, medians, inter-quartile ranges, means, and proportions were used to describe sociodemographic, baseline, and follow-up characteristics.

Dividing the number of participants who experienced virological failure by the total number of participants included in the analysis (257) yielded the proportion of participants who experienced virological failure. We determined the number of times each individual’s viral load exceeded the LOD (>1,000 copies/mL) during the follow-up period. Then, we calculated the average number of these occurrences across all participants who experienced virological failure.

Factors associated with virological first-line ART failure were identified using bivariable and multivariable binary logistic regression models. Variables with a *p*-value ≤ 0.25 in the bivariable binary logistic regression model were included in the multivariable binary logistic regression model to identify factors associated with virological first-line treatment failure. A significance level of 0.05 was used to guide the interpretation of relationships in the final multivariable model, with odds ratios (ORs) and 95% confidence intervals (CIs) calculated.

After the model was built, post-model estimation diagnostics were performed. The goodness-of-fit (GOF) of the model was assessed using the Hosmer–Lemeshow test. This was performed by dividing the predicted probabilities into declines and computing a Hosmer–Lemeshow chi-squared test that compared the predicted and observed frequencies (24). An analysis was conducted to evaluate and assess the accuracy of the model’s predictions. The area under the receiver operating characteristic (ROC) curve, which ranges from 0 to 1, provides a measure of the model’s ability to discriminate between participants who experience the outcome of interest versus those who do not. A value close to 1 indicates that the model has good predictive ability.

## 3 Results

### 3.1 Sociodemographic characteristics

A total of 257 people living with HIV (PWH) were included in the study; 94.48% of the records were complete. All included participants had been on first-line ART for at least 6 months and had undergone at least two viral load assessments within a three-month interval. Female patients comprised 162 (63.14%) of the participants, and the median age was 35 years (IQR = 28–41). The majority of the participants (95.29%) were from urban areas. Most patients had received formal education (71.55%), (see Table 1).

**Table 1:** Sociodemographic characteristics of the adults who received first-line ART from 2017 to 2020 in West Hararghe (*n* = 257).

Characteristics	Categories	Number	Percent
Age at enrollment (in years)	≥ 35	131	50.97
	25–34	96	37.35
	15–24	30	11.67
Sex	Female	162	63.14
	Male	95	36.86
Address	Rural	12	4.71
	Urban	245	95.29
Marital status^a^	Married	125	48.63
	Widowed	11	4.28
	Divorced	15	5.84
	Never married	101	39.3
Educational status^a^	No formal education	46	17.90
	Primary education	80	31.13
	Secondary education	86	33.46
	Diploma and above	42	16.34
Occupation	Government employee	25	9.72
	NGO employee	16	6.22
	Private organization employee	35	13.61
	Self-employed	55	21.40
	Housewife	28	10.89
	Jobless	74	28.79
	Others	24	9.34

NGO, Non-government organization.

a Missing data: marital status was incomplete for five participants, and educational status for three participants.

### 3.2 Baseline, follow-up, clinical, and programmatic characteristics

BMI measurements at 6, 12, and 24 months were included to assess changes in body composition over time and to identify potential associations with virological failure. By analyzing BMI trends, we can gain insights into the nutritional status of participants and guide interventions.

The median weight of the study participants was 56 kg (IQR = 49 kg – 64.5 kg), with a median BMI of 20.57 at enrollment. The median BMI at the 6th month was 21.50 (IQR = 18.91–24.21), at the 12th month was 22.10 (IQR = 19.36–24.75), and at the 24^th^ month was 22.23 (IQR = 19.33–25.28). Only 42 (18.34%) of the participants were unable to perform their usual work, either inside or outside their house, at the time of enrollment in this study. Of these, 13.97% were ambulatory, 4.37% were bedridden, and 81.66% were able to perform activities of daily living. Similarly, 119 (46.30%) participants were categorized as being at advanced WHO clinical stage III or V of HIV/AIDS.

A total of 166 participants (66.94%) disclosed their HIV-positive serostatus to their family members, including spouses, parents, own children, siblings, relatives, or friends. The majority of the participants were initiated on the TDF + 3TC + EFV regimen (63.43%), regardless of any criteria (46.69%), within a median duration of 5 days (IQR = 0–22) after testing HIV-positive. Only 94 (36.57%) of the patients initiated the regimen TDF + 3TC + DTG, which included the newly introduced drug DTG.

At the 6th month, 30.09% of the participants were at an advanced WHO clinical stage (stage III or IV) of HIV/AIDS, with 12.99% having a non-functional status (9.09% ambulatory and 3.90% bedridden). The mean duration on ART was approximately 30 months (29.66 ± 10.03). In addition, approximately 17.11% of the patients with recorded ART adherence assessments at the 6th month had poor adherence, and 33.19% of the patients had a recorded history of missed ART doses.

In this study, isoniazid (INH) prophylaxis was typically recommended for the individuals with a positive TST and a negative chest X-ray. Cotrimoxazole preventive therapy (CPT) was recommended for the individuals with a CD4 count below 200 cells/mm^3^.

A total of 166 patients (64.73%) completed isoniazid (INH) prophylaxis, while only 37.74% completed cotrimoxazole preventive therapy (CPT). In addition, 31 patients (11.95%) were diagnosed with TB at the time of follow-up (Table 2).

**Table 2:** Baseline and follow-up clinical and programmatic characteristics of the adults who received first-line ART in West Hararghe from 2017 to 2020 (*n* = 257).

Baseline characteristics	Categories	Number	Percent
BMI at enrollment	18.5–24.99	138	53.69
	< 18.5	82	31.90
	> 24.99	37	14.39
Functional status at enrollment	Ambulatory	36	13.97
	Bedridden	11	4.37
	Working	210	81.66
WHO clinical stage at enrollment	Stage I and II	138	53.69
	Stage III and IV	119	46.30
CD4 count at enrollment (cells/μL)	< 251	71	27.62
	251–500	145	59.92
	≥ 500	41	15.95
HIV-positive serostatus disclosure^a^	Yes	166	66.94
	No	82	33.06
Eligibility criteria for ART initiation	Regardless of any criteria	120	46.69
	With criteria	137	53.30
Duration of ART initiation after HIV diagnosis (days)	Early (≤ 30 days)	160	62.25
	Late (> 30 days)	97	37.75
ART regimen initiated	TDF + 3TC + EFV	163	63.43
	TDF + 3TC + DTG	94	36.58
Follow-up characteristics			
BMI at 6th month (kg/m^2^)	18.5–24.99	160	62.13
	< 18.5	50	19.57
	> 24.99	47	18.3
Functional status at 6th month	Ambulatory	23	9.09
	Bedridden	10	3.9
	Working	224	87.01
WHO clinical Stage at 6th month^a^	Stage I and II	178	69.90
	Stage III and IV	77	30.09
CD4 count (cells/mL) at 6th month	< 251	9	3.57
	251–500	151	58.56
	≥ 500	97	37.86
TB diagnosis	Negative	226	88.05
	Positive	31	11.95
TB prophylaxis	INH completed	166	64.73
	On INH /discontinued	53	20.75
	INH never administered	38	14.52
Cotrimoxazole preventive therapy (CPT)	Completed CPT	97	37.74
	On CPT/discontinued	71	27.81
	CPT never administered	89	34.63
Adherence (6th month)	Good	198	77.19
	Fair	15	5.70
	Poor	44	17.11
History of missed ART doses	No	172	66.81
	Yes	85	33.19
ART regimen first substitution	Yes	145	56.47
	No	112	43.53
Duration on first-line ART (in months)	≤ 24	106	41.2
	> 24	151	58.75

a Missing data: HIV-positive serostatus disclosure was incomplete for nine participants, and WHO clinical stage at the 6th month was incomplete for two participants.

### 3.3 Virological first-line ART failure

More than 1 in 10 patients experienced virological failure on first-line ART (11.67, 95%CI = 7.69, 15.80) (Table 3).

**Table 3:** Sociodemographic and clinical factors significantly associated with virological failure on first-line ART among the adults enrolled from 2017 to 2020 in West Hararghe (*n* = 257).

Variable	Classification	Suppressed *N* (%)	Failed *N* (%)	AOR (95%CI)
Sex	Female	146 (90.68)	16 (9.32)	1
	Male	81 (85.26)	14 (14.73)	1.852 (0.566, 6.061)
Age at enrollment	≥ 35	119 (91.27)	12 (8.73)	1
	25–34	84 (87.10)	12 (12.90)	1.530 (0.377, 6.214)
	15–24	24 (80)	6 (20)	2.126 (0.302, 14.970)
Clinical characteristics at enrollment				
BMI at enrollment (kg/m^2^)	18.5–24.99	130 (93.75)	8 (6.25)	1
	< 18.5	64 (81.54)	18 (18.46)	3.717 (1.051, 13.139)
	> 24.99	33 (89.19)	4 (10.81)	1.322 (0. 108, 16.210)
Serostatus disclosure	Yes	153 (92.17)	13 (7.83)	1
	No	65 (79.27)	17 (20.73)	4.453 (1.340, 14.793)
WHO stages at enrollment	I & II	127 (92.03)	11 (7.97)	1
	III & IV	100 (83.58)	19 (16.42)	3.036 (0.898, 10.267)
Duration of ART initiation (in days)	Early (≤ 30 days)	148 (92.5)	12 (7.5)	1
	Late (> 30 days)	79 (81.44)	18 (18.56)	4.249 (1.164, 15.512)
Clinical characteristics during follow-up				
History of missed ART doses	No	159 (92.50)	13 (7.50)	1
	Yes	68 (79.75)	17 (20.25)	3.156 (1.007, 9.891)
DTG-based regimen	No	93 (83.03)	19 (16.96)	1
	Yes	134 (92.41)	11 (7.59)	0.275 (0. 085, 0.895)
Duration on ART (in months)	≤ 24	100 (94.34)	6 (5.66)	1
	> 24	127 (84.56)	23 (15.44)	2.821 (0.775, 10.273)

### 3.4 Factors associated with Virological ART failure

Potential factors associated with virological failure on first-line ART were identified using bivariable logistic regression (*p* ≤ 0.25) and were further analyzed using multivariable logistic regression. In the bivariable logistic regression analysis, sex, age at enrollment, baseline BMI, HIV-positive serostatus disclosure, baseline WHO clinical stage, duration before ART initiation after HIV diagnosis, a history of missed ART doses, and substitution of the DTG-based ART regimen were selected for multivariable logistic regression. Finally, five predictors—baseline BMI, HIV-positive serostatus disclosure, duration before ART initiation after HIV diagnosis, a history of missed ART doses, and substitution of the DTG-based ART regimen—were found to have a statistically significant association with virological failure on first-line ART during the multivariable logistic regression analysis.

The multivariable logistic regression analysis revealed that the patients who did not disclose their HIV-positive serostatus showed higher odds (AOR = 4.453: 1.340, 14.793) of virological ART failure as compared to the patients who disclosed their status. In addition, the undernourished patients (baseline BMI ≤ 18.5) had higher odds (AOR = 3.717: 1.051, 13.139) of virological ART failure than the patients with a considerably normal nutritional status (BMI = 18.5–24.99).

The PWH who initiated ART > 30 days after HIV diagnosis showed higher odds of virological ART failure than those who initiated ART ≤ 30 days after the diagnosis. In the same way, the participants who initiated the newly recommended DTG-based regimen or whose ART regimen was substituted with the new first-line ART regimen (TDF + 3TC + DTG) showed lower odds (AOR = 0.275: 0. 085, 0.895) of virological ART failure compared to the patients on other regimens. After ART initiation, the patients with a recorded history of missed ART doses had higher odds (AOR = 3.156: 1.007, 9.891) of virological ART failure compared to their counterparts (Table 3).

**Figure 2:**
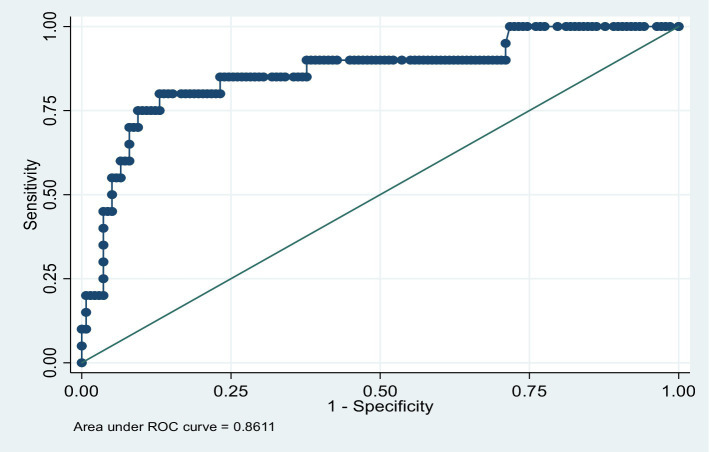
ROC showing the good predictive ability of the final model.

Finally, after the model was built, post-model estimation diagnostics were performed. The GOF test for the virological outcome logistic model showed acceptable results (Pearson chi-squared (10) = 10.68, *p* = 0.3828). The ROC also showed good predictive ability for the model (86.11% area under the curve), with an acceptable classification rate (correctly classified = 88.61%) (Figure 2).

## 4 Discussion

Despite the successful implementation of HIV treatment programs for PWH, we reported relatively high rates of treatment failure among the PWH who received first-line ART regimens in West Hararghe. This incidence of treatment failure was significantly associated with HIV-positive serostatus non-disclosure, undernutrition (baseline BMI < 18.5), a history of missed ART doses (any missed daily dose), late (> 30 days) initiation of ART after HIV diagnosis, and the use of non-DTG-based ART regimens. Nutritional status (underweight) and HIV serostatus non-disclosure were baseline patient characteristics that negatively affected virological failure. Interruptions in ART doses (inadequate adherence) were another identified risk factor that might result in drug resistance, thereby increasing the odds of virological failure. The odds of virological failure were lower for the patients who initiated ART within 30 days of HIV diagnosis and for those on the DTG-based regimen, compared to their counterparts.

The present findings revealed that more than one-tenth of the enrolled adults experienced virological failure on first-line ART—an important clinical concern in West Hararghe—despite the 95–95–95 national action plan aimed at achieving over 95% viral suppression, as recommended by UNAIDS (25). This finding is more or less similar to previous study reports from Ethiopia (11.5%) and Uganda (11%) (16, 26, 27). Other similar studies reported virological failure rates of 10.24% (28), 13% (29) from Ethiopia, and 9% across four African countries (30). Previous studies conducted in Ethiopia (14, 22, 31), India (32), China (33), and Swaziland (34) also reported higher rates than those observed in the present study. These slight variations might be due to differences in socioeconomic status (35, 36), study populations (36), sample sizes, availability of medical services, and the methods used to investigate virological failure, all of which may influence the detection of treatment failure (37).

This study also revealed that HIV serostatus disclosure decreases the odds of virological failure. It is well established that non-disclosure of HIV serostatus negatively impacts ART adherence [(38, 39), which can subsequently lead to virological failure (38, 40). Unlike the findings of this study, a previous Ethiopian study reported a higher incidence of virological failure among adults who had disclosed their HIV-positive status, possibly due to stigma and discrimination (41). HIV-positive serostatus non-disclosure and ART interruption might result in the loss of opportunities to suppress viral replication, ultimately leading to virological failure (42, 43). If not handled properly, disclosure of HIV-positive status to friends and family may lead to a loss of support (44) and HIV serostatus-related discrimination (45). Similarly, missing ART doses can increase the odds of virological failure, as shown in this study and studies conducted elsewhere (30, 46). Missed ART doses (inadequate ART adherence) may lead to treatment failure, possibly due to the development of acquired drug resistance (47, 48).

In this study, the undernourished patients (baseline BMI < 18.5 kg/m^2^) showed higher odds of virological failure than the patients with normal baseline nutritional status (baseline BMI = 18.5–24.99 kg/m2) (AOR = 3.717: 1.051, 13.139). This is comparable to a previous study conducted in Ethiopia (10, 49). Other studies from Ethiopia (10, 19, 50, 51) showed similar findings. A possible reason for this could be that patients with low BMI have poor nutritional status, which leads to weakened immunity, a blunted immune response, and poor virological outcomes (52–54).

Late ART initiation (initiation within 30 days of HIV diagnosis) was significantly associated with increased odds of virological failure. This finding is consistent with reports from other studies, which suggest that early ART initiation may benefit HIV patients by enhancing viral suppression and survival (55–57). This study suggests that accelerated ART initiation generally leads to improved virological outcomes, thus supporting the recommendation in the Ethiopian ART guidelines for accelerated ART initiation.

Factors associated with virological failure were explored, including the type of first-line ART regimen initiated. DTG-based regimes were statistically associated with lower rates of virological failure compared to other first-line ART regimens. This finding is in line with reports from studies that found evidence of virological suppression being associated with regimens containing DTG (58, 59). Based on the WHO recommendation (3), Ethiopia’s ART guidelines were revised in August 2018 to prioritize the first-line use of integrase inhibitors (DTG) over EFV (21). Non-nucleoside reverse-transcriptase inhibitor drug resistance, particularly to EFV, is an important key factor driving the switch to DTG (60), while DTG-based regimens are potent treatment options (59, 61). Poor adherence is one of the factors that contribute to drug adaptability and resistance development (62).

### 4.1 Strengths and limitations of the study

Since the implementation of the test-and-treat approach, along with the recent use of DTG for first-line ART and routine viral monitoring, variables not reported in other similar studies, to the best of the author’s knowledge, were considered for verification. This study has some limitations. It was conducted at a single site, which might have limited the generalizability of the findings to other settings. In addition, due to the small sample size, certain variables might have been either over- or under-estimated in their impact on viral suppression. Finally, the design of the study did not include viral genotyping to confirm the presence of resistance or determine the incidence rate and hazard ratio for direct measurement of risk. Although these are important programmatic interests, they would require a cohort study, which is beyond the scope of this research.

## 5 Conclusion

Virological first-line treatment failure was significantly higher in West Hararghe. Hindering factors identified in this study, such as undernutrition, non-disclosure serostatus, and interruptions in ART doses, may contribute to the higher burden of HIV treatment failure. The study findings also emphasize the early initiation of ART (preferably DTG-based regimens) with frequent virological monitoring to sustain the ART treatment program. All stakeholders in the West Hararghe HIV control program need to review the recommended standards of care and interventions to address the identified contributing factors. Healthcare providers should take into account the patient’s nutritional status and serostatus disclosure when enrolling patients. In conjunction with the early initiation of ART, a comprehensive nutritional assessment and supplemental nutritional therapy for undernutrition could be important. Future studies should be conducted using prospective and qualitative designs to identify factors influencing virological treatment outcomes among adults on ART.

## Data Availability

The original contributions presented in the study are included in the article/Supplementary material, further inquiries can be directed to the corresponding author.

## Glossary

ABC: Abacavir
AOR: Adjusted Odd Ratio
ART: Anti-Retroviral Therapy
ARV: Antiretroviral drugs
BMI: Body Mass Index
AZT: Azidothymidine (also known as Zidovudine, abbreviated as ZDV)
CD4 cells: Cluster for Differentiation 4 cells, type of T-lymphocyte bearing CD4
CPT: Cotrimoxazole preventive therapy
DTG: Dolutegravir
EAC: Enhanced Adherence Counseling
EFV: Efavirenz
EPHI: Ethiopian Public Health Institute
MUAC: Middle Upper Arm Circumference
NNRTI: Nonnucleoside Reverse Transcriptase Inhibitor
NVP: Nevirapine
PWH: People with HIV
3TC: Lamivudine
TDF: Tenofovir
TB: Tuberculosis
WHO: World Health Organization
