# Supplementary material for: Virological treatment failure and associated factors among adults on first-line antiretroviral therapy in West Hararghe, Ethiopia
Source: Front Public Health. 2025 Jun 2;13:1440504. doi: 10.3389/fpubh.2025.1440504 (PMC12171124; doi:10.3389/fpubh.2025.1440504)
Supplement: Supplementary file 1 [file Table_1.DOCX]

| Factors associated with virological failure | % of outcome among unexposed | AOR | Sample size adding 20% dropout rate | Reference |
| --- | --- | --- | --- | --- |
| Poor adherence | 38% | 6.37 | 242 | Nega, et al. 2020 |
| C CD4 count <500 | 22.45% | 4.78 | 272 | Nega, et al. 2020 |
| Duration on ART (6-24 Months) | 2.04% | 0.48 | 191 | Nega, et al. 2020 |

**Supplementary Table 1**

**Sample size for the factors associated with virological failure**
