# Supplementary material for: Virological treatment failure and associated factors among adults on first-line antiretroviral therapy in West Hararghe, Ethiopia
Source: Front Public Health. 2025 Jun 2;13:1440504. doi: 10.3389/fpubh.2025.1440504 (PMC12171124; doi:10.3389/fpubh.2025.1440504)
Supplement: Supplementary file 2 [file Data_Sheet_1.PDF]

Supplement Table 2. Factors Associated with Virological First-line ART Failure

| Variable | Classification | Suppressed N (%) | Failed N (%) | AOR (95%CI) | P-value |
|---|---|---|---|---|---|
| **Sex** | Female | 146 (90.68) | 15 (9.32) | 1 | |
| | Male | 80 (85.11) | 14 (14.89) | 1.852 (0.566, 6.061) | 0.06 |
| **Age at Enrollment** | ≥ 35 | 115 (91.27) | 11 (8.73) | 1 | |
| | 25 – 34 | 81 (87.10 ) | 12 (12.90) | 1.530 (0.377, 6.214) | 0.056 |
| | 15 - 24 | 24 (80) | 6 (20) | 2.126 (0.302, 14.970) | 0.082 |
| **Clinical Characteristics at Enrollment** | | | | | |
| **BMI at Enrollment ($kg/m^2$)** | 18.5 - 24.99 | 120 (93.75) | 8 (6.25) | 1 | |
| | < 18.5 | 53 ( 81.54) | 12 (18.46) | 3.717 (1.051, 13.139) | 0.02* |
| | > 24.99 | 33 (89.19) | 4 (10.81) | 1.322 (0. 108, 16.210) | 0.074 |
| **Serostatus Disclosure** | Yes | 153 (92.17) | 13 (7.83) | 1 | |
| | No | 67 (81.71) | 15 (18.29) | 4.453 (1.340, 14.793) | 0.001* |
| **WHO Stages at Enrollment** | I & II | 122 (89.71) | 14 (10.29) | 1 | |
| | III & IV | 56 (83.58) | 11 (16.42) | 3.036 (0.898, 10.267) | 0.057 |
| **Duration of ART Initiation (in Days)** | early (≤ 30 days) | 137 (93.20) | 10 (6.80) | 1 | |
| | late (> 30 days) | 72 (80.00) | 18 (20.00) | 4.249 (1.164, 15.512) | 0.02* |
| **Clinical Characteristics During Follow-up** | | | | | |
| **History of Missed ART Doses** | No | 148 ( 93.08 ) | 11 (6.92) | 1 | |
| | Yes | 63 (79.75) | 16 (20.25) | 3.156 (1.007, 9.891) | 0.03 * |
| **DTG Based Regimen** | No | 81 (81.82) | 18 (18.18) | 1 | |
| | Yes | 147 (93.04) | 11 (6.94) | 0.275 (0. 085, 0.895) | 0.001 * |
| **Duration on ART (in Months)** | ≤ 24 | 100 (94.34) | 6 (5.66) | 1 | |
| | > 24 | 126 (84.56) | 23 (15.44) | 2.821 (0.775, 10.273) | 0.53* |

*****- statistically significant at p-value < 0.05.
